# Transcriptome profiling of the floating-leaved aquatic plant *Nymphoides peltata* in response to flooding stress

**DOI:** 10.1186/s12864-017-3515-y

**Published:** 2017-01-31

**Authors:** Jinwei Wu, Hua-Bin Zhao, Dan Yu, Xinwei Xu

**Affiliations:** Department of Ecology, College of Life Sciences, Wuhan University, 299 Bayi Road, Wuhan, 430072 China

**Keywords:** *Nymphoides peltata*, Transcriptome, Flooding stress, qRT-PCR

## Abstract

**Background:**

Waterlogging or flooding is one of the most challenging abiotic stresses experienced by plants. Unlike many flooding-tolerant plants, floating-leaved aquatic plants respond actively to flooding stress by fast growth and elongation of its petioles to make leaves re-floating. However, the molecular mechanisms of this plant group responding to flood have not been investigated before. Here, we investigated the genetic basis of this adaptive response by characterizing the petiole transcriptomes of a floating-leaved species *Nymphoides peltata* under normal and flooding conditions.

**Results:**

Clean reads under normal and flooding conditions with pooled sampling strategy were assembled into 124,302 unigenes. A total of 8883 unigenes were revealed to be differentially expressed between normal and flooding conditions. Among them, top ranked differentially expressed genes were mainly involved in antioxidant process, photosynthesis process and carbohydrate metabolism, including the glycolysis and a modified tricarboxylic acid cycle – alanine metabolism. Eight selected unigenes with significantly differentiated expression changes between normal and flooding conditions were validated by qRT-PCR.

**Conclusions:**

Among these processes, antioxidant process and glycolysis are commonly induced by waterlogging or flooding environment in plants, whereas photosynthesis and alanine metabolism are rarely occurred in other flooding-tolerant plants, suggesting the significant contributions of the two processes in the active response of *N. peltata* to flooding stress. Our results provide a valuable genomic resource for future studies on *N. peltata* and deepen our understanding of the genetic basis underlying the response to flooding stress in aquatic plants.

**Electronic supplementary material:**

The online version of this article (doi:10.1186/s12864-017-3515-y) contains supplementary material, which is available to authorized users.

## Background

Understanding how individuals respond to ever-changing environments is of fundamental importance in all organisms. Plants encounter various biotic and abiotic stresses throughout their life span. One of the most dramatic abiotic stresses is flooding [1]. Plants cannot actively escape a flooding environment due to their immobile character [2], and many plants are injured or killed by flooding events due to oxygen shortage in their cells [3, 4]. However, over the long period of adaptive evolution, plants have evolved the capacity to survive flooding habitats via escape phenotypes, e.g., the shoot elongation, the formation of aerenchyma, and the induction of gas films [5]. Recently, the molecular mechanisms of physiology and metabolic modulation behind these adaptive traits have been investigated in crops and wetland plants [6–10]. However, few such studies have been conducted on true aquatics, floating-leaved or submerged aquatic plants [11], which are specialized to life in water and likely have different responding mechanisms to flooding compared with other plants.

The fringed water lily *Nymphoides peltata* (S. G. Gmelin) Kuntze is a typical floating-leaved plant with a widespread distribution in temperate and subtropical regions of Eurasia [12]. *Nymphoides peltata* usually roots in the bottom mud and maintains its leaves afloat on the water surface with the connection of petioles. When subjected to flooding, the leaves of *N. peltata* can rapidly reach the water surface by rapid elongation of the petioles [13, 14], an ability that is also present in many other floating-leaved plants [15]. This trait makes *N. peltata* an ideal aquatic species for investigating the molecular mechanisms of plants to avoid submergence stress. Previous study showed that ethylene played a major role in the case where submergence promotes petiole elongation in *N. peltata* [13]. which is similar with many aquatic or flooding-tolerant species, e.g., *Rumex palustris* [6] and rice [10]. However, the molecular mechanisms of flood-adaption in *N. peltata* still remain unclear. As a non-model plant, genomic information of *N. peltata* is scarce, except for the development of several molecular markers [16, 17], which hinders the exploration of the underlying flood-adaption mechanisms in this plant. With the emergence of next-generation sequencing technologies, a new technology RNA-Seq (RNA sequencing) independent of genetic background has been developed [18, 19]. Recently, RNA-Seq has been utilized to elucidate the response of non-model plants to various environmental stresses, including flooding and waterlogging [20–23].

In this study, we examined the global gene expression changes of *N. peltata* under both normal and flooding conditions using Illumina RNA-Seq technology. The results provide a comprehensive view of the complex molecular events involved in the response of floating-leaved plants to flooding stress and expand our understanding of response to flooding stress.

## Results

### Illumina sequencing and assembly

In total, 78,037,588 and 103,266,542 clean reads were obtained from the untreated sample (US) and the treated sample (TS), respectively (Table 1). Assembly of those reads from US and TS separately generated 87,673 and 95,372 unigenes, respectively (Table 1). The strategy of pooling all clean reads from US and TS together generated 124,302 unigenes with a N50 length of 1449 bp after assembly (Table 1). The number of unigenes from the pooled strategy was larger than those from separate assemble because some unigenes with low expression levels generated with the pooled strategy cannot be found when using strategy of separate assemble due to their less reads. Among these 124,302 unigenes, the total number of unigenes longer than 500 bp was 56,943, accounting for 45.81% of all unigenes (Additional file 1). The detailed length distribution of 124,302 unigenes predicted from the pooled assembly can be also found in Additional file 1.

**Table 1:** Overview of transcriptome sequencing and unigene annotations

	Untreated sample (No flooding stress)	Treated sample (Under flooding stress)	Total
Raw reads	87,520,118	115,308,872	
Clean reads	78,037,588	103,266,542	
Q20 (%)^a^	95.31	95.63	
Unigene number^b^	87,673	95,372	124,302
Unigene N50 (bp)^b^	1195	1140	1449
NR database^b^	37,697	42,654	53,744
Swissprot database^b^	26,086	31,422	37,556
GO deatabase^b^	22,602	25,733	30,326
KEGG database^b^	8409	10,048	11,408
COG database^b^	34,413	39,180	48,156
Total annotated^b^	43.81%	45.11%	43.86%

^a^The percentage of sequences with an error rate < 1%

^b^These statistics were based on an assembly of all clean reads from both treated and untreated samples

### Gene annotation and functional classification

Unigenes generated using the pooled strategy were used for further analysis. Among these 124,302 unigenes, a total of 53,870 (43.34%) unigenes were annotated (Table 1). According to the NCBI non-redundant proteins (NR) annotation, 53,744 (43.24%) unigenes had homologous proteins in the NR protein database (Table 1). Meanwhile, unigenes were also matched with GO category, and 30,326 (24.40%) unigenes were assigned to one or more GO terms (Table 1). Using GO annotation, the functions of the unigenes were divided into three categories: biological process, cellular component, and molecular function. To obtain a detailed view of the GO classification, each GO category was further clustered to its parent term (Additional file 2). The results indicate that most of the sequenced genes were responsible for fundamental biological regulation and metabolism.

Furthermore, the possible functions of 124,302 unigenes were predicted using searches against the Cluster of orthologous groups (COG) database as well as Swiss-Prot Protein Sequence (Swissprot) database. A total of 48,156 unigenes were matched with the COG database (Table 1) and classified into 25 specific categories (Additional file 2). The “General function prediction only” was the largest group (20.08%), followed by “Posttranslational modification, protein turnover, chaperones” (9.99%) and “Signal transduction mechanisms” (9.21%) (Additional file 2). We also obtained 37,556 hits when searched against Swissprot protein database with an E-value of 1.0E-5, covering 31.62% of all unigenes (Additional file 3). All the deduced protein sequences corresponding to the predicted coding DNA sequences (CDS) were listed in Additional file 4. Kyoto encyclopedia of genes and genomes (KEGG) pathway analysis was also conducted to predict the function of the unigenes during the development process. In total, 11,409 unigenes were found to be involved in 244 pathways (Table 1).

We also used the phytozome database (http://www.phytozome.net/) to annotate unigenes. Similar as the NR database, the percent of annotated unigenes was about 43% (Additional file 5), which is much lower than a normal annotation percentage 70% in other plants. To examine whether these unannotated unigenes were non-coding RNA genes or lineage-specific genes, we predicted sequences of the unannotated unigenes and found that most of them have predicted CDS. We further used the unannotated unigenes as queries to search against the plant noncoding RNAs database and found that only a small proportion of unigenes matched the sequences in the noncoding RNAs database (Additional file 5). Therefore, most of the unannotated unigenes were inferred to be *Nymphoides peltata* lineage-specific genes.

### Comparison between treated and untreated samples

**Fig. 1:**
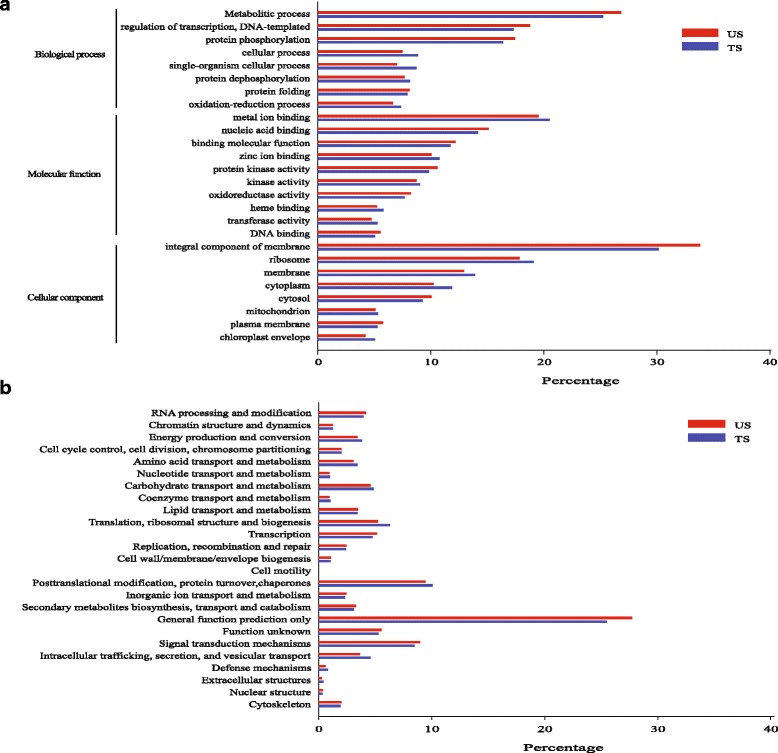
GO (Gene ontology) and COG (Cluster of orthologous groups) annotation. **a** GO annotation of the assembled unigenes for the treated sample (TS) and the untreated sample (US) separately, the functions of the unigenes were divided into three categories. **b** Information of COG classification for the treated sample (TS) and the untreated sample (US) separately, the unigenes were mainly clustered into 25 components

Functional annotation and COG classification for the separately assembled unigenes for samples TS and US was also conducted after annotation of unigenes using the pooled strategy, the results were shown in Fig. 1. The number of unigenes in each category for GO and COG annotation showed parallel proportion between these two samples, indicating that transcriptomes of sample TS and US were comparable.

Furthermore, we listed the top 20 ranked unigenes with high expression level in sample TS and the corresponding unigenes in sample US (Table 2). Among these 20 unigenes, 10 were DEGs, which encoded proteins mainly involved in osmoregulation (e.g., aquaporin and osmotin-like protein) and proline-rich protein (Table 2). The former is very helpful in the regulation of water homeostasis and water transport, and the latter is a cell wall protein of plant regulating plant wound and defense responses. As for the other 10 unigenes, they were mainly involved in photosynthesis process (e.g., ribulose bisphosphate carboxylase and chlorophyll a-b binding protein) and antioxidant process (e.g., peroxidase and glutamine synthetase cytosolic isozyme) (Table 2).

**Table 2:** The top 20 ranked unigenes (based on expression level) in the TS sample and the corresponding unigenes in the US sample

Unigene ID	FPKM		Log_2_FC	Swissprot annotation
	US	TS		
CL1448Contig1*	4098.9	6615.6	0.6906	Putative uncharacterized protein ART2
con2.comp50853_c1_seq1*	2359.4	5061.6	1.1012	Ribulose bisphosphate carboxylase
con2.comp55521_c1_seq2*	2683.5	2716.9	0.0178	Chlorophyll a-b binding protein
**CL19733Contig1**	62.02	2388.1	5.2670	Aquaporin TIP1-1
**d2.comp66814_c0_seq1**	405.58	2025.4	2.3201	Homocysteine methyltransferase
con2.comp46855_c0_seq1	563.36	2014.8	1.8385	Chlorophyll a-b binding protein
**con2.comp56741_c0_seq1**	272.93	1970.4	2.8519	Adenosyl homocysteinase sahh
**CL21Contig8**	281.14	1902.9	2.7588	Pistil-specific extensin-like
**d2.comp52786_c0_seq1**	96.70	1769.6	4.1938	Osmotin-like protein osml13
**CL14756Contig1**	30.91	1628.7	5.7195	14 kda proline-rich protein
con2.comp43560_c0_seq1*	1158.0	1462.4	0.3367	Lipid-transfer protein dir1
d2.comp33048_c0_seq1	766.12	1450.9	0.9213	Chlorophyll a-b binding protein
**con2.comp58109_c0_seq5**	49.71	1442.6	4.8590	Proline-rich protein
CL10800Contig1	414.22	1422.8	1.7803	Isoflavone reductase-like protein
d2.comp65178_c0_seq2*	802.5	1416.2	0.8195	Chlorophyll a-b binding
d2.comp61695_c0_seq1*	1033.1	1370.1	0.4073	Glutamine synthetase cytosolic isozyme
**con2.comp53151_c0_seq8**	252.52	1350.1	2.4186	Aquaporin TIP1-1
**d2.comp59904_c0_seq3**	129.21	1349.1	3.3842	Proline-rich protein
d2.comp54069_c0_seq1	413.02	1343.1	1.7013	Peroxidase 12
**CL68Contig3**	68.81	1341.1	4.2847	Chlorophyll a-b binding protein

Note: *US* Untreated Sample, *TS* Treated Sample. FPKM represents unigene expression level that is normalized by FPKM (Fragments per Kilobase per Million mapped reads) approach. The six unigenes denoted with an asterisk (*) indicated highly expressed unigenes in both untreated and treated samples. The unigenes in bold are differentiated expressed genes (DEGs) between US and TS sample

### Analysis of differentially expressed genes

After calculating the unigene expression level, with an FDR (false discovery rate) of 0.05 and |log_2_Fold Change| ≥ 1 as a cutoff, a total of 8883 (6401 up-regulated and 2482 down-regulated) unigenes were revealed to be significantly differentially expressed between the treated and untreated samples. The top 20 ranked differentially expressed unigenes were identified. Among them, most genes were involved in basic cell component (e.g., proline-rich protein and ribosomal protein), photosynthesis (e.g., chlorophyll a-b binding protein, chloroplastic glyceraldehyde 3-phosphate dehydrogenase, and ribulose bisphosphate carboxylase) and antioxidant process (e.g., L-ascorbate oxidase homolog, ferric reduction oxidase, and peroxidase) (Table 3).

**Table 3:** The top 20 ranked most differentially expressed unigenes between normal and flooding conditions

Unigene ID	Log_2_FC	FDR	Swissprot annotation
d2.comp62123_c2_seq4	13.94	5.99E-20	Chlorophyll a-b binding protein
d2.comp52108_c0_seq2	13.78	9.43E-20	Auxin-induced in root cultures protein
d2.comp54965_c0_seq2	13.09	5.46E-18	Proline-rich protein 4
d2.comp53053_c0_seq1	12.78	2.03E-17	Kirola
d2.comp66621_c0_seq4	12.71	2.94E-17	Protein da1-related 1
d2.comp60426_c0_seq5	12.68	3.27E-17	Benzyl alcohol 0-benzoyltransferase
d2.comp57055_c0_seq2	12.16	7.80E-16	mitochondrial chaperone bcs1
d2.comp61187_c1_seq1	11.68	1.84E-14	Glyceraldehyde 3-phosphate dehydrogenase chloroplastic
d2.comp56332_c0_seq2	11.58	3.11E-14	Heat shock cognate 70 kda protein 2
d2.comp58962_c0_seq1	11.57	3.22E-14	Cluminal-binding protein 4
d2.comp61381_c0_seq1	11.42	6.45E-14	Heat shock cognate protein 80
d2.comp63783_c0_seq1	11.37	9.00E-14	L-ascorbate oxidase homolog
d2.comp62781_c0_seq1	11.29	1.42E-13	Probable ferric reduction oxidase 1
d2.comp57757_c0_seq1	11.28	1.45E-13	40s ribosomal protein s2-2
d2.comp62067_c0_seq1	11.27	1.50E-13	Carrier protein chloroplastic
d2.comp59687_c0_seq1	11.12	3.92E-13	Uncharacterized protein At1g08160
d2.comp54780_c1_seq1	11.11	4.24E-13	Elongation factor 1-alpha
d2.comp57273_c0_seq1	11.03	6.61E-13	Chlorophyll a-b binding protein
d2.comp54437_c2_seq1	11.02	6.61E-13	Peroxidase 27
d2.comp55364_c0_seq2	11.00	7.07E-13	Ribulose bisphosphate carboxylase

Note: Log_2_FC was estimated by the difference between Log_2_(FPKM of TS sample) and Log_2_(FPKM of US sample). FDR: *p* value corrected by false discovery rate at 5%

**Fig. 2:**
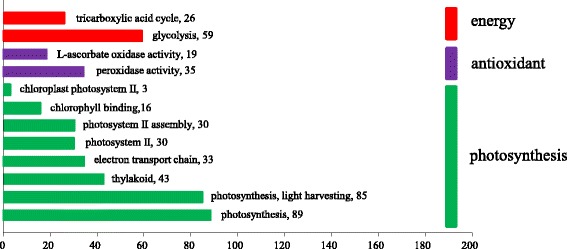
GO enrichment analysis of putatively selected GO terms of DEG functions. *DEG* differentially expressed gene. Gene number is shown next to each GO term

GO enrichment analysis of DEGs indicated that 153 GO terms were significantly enriched with the criteria of FDR < 0.01. Of them, 71 terms were enriched in the category of biological process, 52 in molecular function, and 30 in cellular component (Additional file 6). Further analysis showed that GO terms related to three aspects (energy, antioxidant, photosynthesis) were significantly important (Fig. 2).

**Fig. 3:**
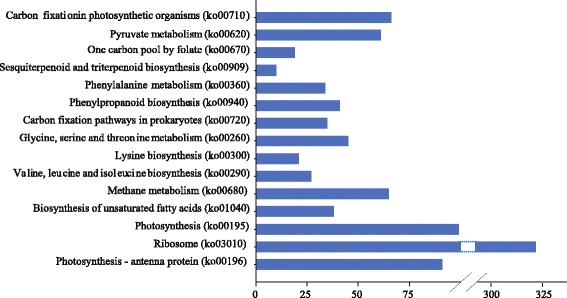
Significantly enriched KEGG pathways of DEG functions. *DEG* differentially expressed gene

KEGG pathway analysis of the DEGs indicated that various genes were working together to execute functions. Overall, the most significant pathways were Ribosome pathway (ko03010) with 315 DEGs enriched, followed by pathways of Photosynthesis (ko00195, 99) and Photosynthesis - antenna protein (ko00196, 91) (Fig. 3). Further analysis showed that pathways involved in energy metabolism, antioxidant process, and photosynthesis process were also present (Fig. 3).

### Transcriptome changes of *Nymphoides peltata* and five other plants in response to flooding

To gain a broad picture of plant responses to flooding, we listed transcriptome changes of *Nymphoides peltata* and five other plants to cope with flooding in Table 4. It appears that common responses to flooding in transcriptome were involved in glycolysis, the antioxidant process, the fermentation and the present of group VII ethylene response factor transcription factors (GVIIERFs) (Table 4). By contrast, expression changes of the alanine metabolism under submergence were only observed in *N. peltata* and *Lotus japonicas* (Table 4). As a species of true aquatic plant, *N. peltata* remained active photosynthesis under submergence, which has not been reported in other plants (Table 4).

**Table 4:** Transcriptome changes of *Nymphoides peltata* (this study) and five other plants in response to flooding

Species	GVIIERFs	Hormone	Energy sources	ROS scavenging enzymes	Fermentation	Alanine metabolism	Photosynthesis	References
*Nymphoides peltata*	present	Ethylene IAA, ABA	Glycolysis, Modified TCA	GST, GPX, APX, SOD	ADH, LDH	AlaAT, AspAT	Increased	(this study, [13])
*Arabidopsis thaliana*	present	Ethylene IAA	Glycolysis	CSD2, GPX, APX	ADH, PDC	AlaAT	unexamined	[5, 47, 50, 61, 62]
*Oryza sativa*	present	Ethylene GA, ABA	Glycolysis	APX, GR, GST, SOD	ADH, PDC	unexamined	unexamined	[10, 62–65]
*Rumex palustris*	present	Ethylene ABA, GA	Glycolysis	GPX, GSH	ADH	unexamined	unexamined	[22]
*Lotus japonicus*	unexamined	Ethylene ABA	Glycolysis, Modified TCA	GST, SOD	ADH, PDC, LDH	AlaAT, AspAT	unexamined	[49, 66]
*Taxodium mucronatum × T. distichum*	unexamined	unexamined	Glycolysis	Peroxidase	ADH, LDH, PDC	unexamined	unexamined	[23]

### Molecular adaptation of aquatic plants compared with non-aquatic plants

To identify molecular adaptation in aquatic plants and help understand flooding response in plants, we applied a likelihood approach to detect molecular adaptation by estimating the ratio (ω) of nonsynonymous to synonymous substitution rates. Our dataset contained three aquatic plants and five non-aquatic plants. Within the aquatic plants, one (*Utricularia gibba*) has a draft genome, and the remaining two (*Ranunculus bungei*, and *Nymphoides peltata*) have transcriptome data. By contrast, all five non-aquatic plants (*Arabidopsis thaliana*, *Oryza sativa*, *Solanum lycopersicum*, *Daucus carota*, and *Cucumis sativus*) possess available genome sequences. A total of 5319 one-to-one orthologous genes were identified in our dataset. Using an established species tree from the Angiosperm Phylogeny Website (http://www.mobot.org/MOBOT/research/APweb/), we tested the possibility of differential selection between aquatic and non-aquatic plants, by comparing a one-ratio model with a two-ratio model. The one-ratio model assumes a uniform ω ratio across all plants, while the two-ratio model assumes two ω ratios: all non-aquatic plants have ω_0_, whereas all aquatic plants have ω_1_. Our analyses yielded 18 genes with significantly higher evolutionary rates in aquatic plants (Additional file 7), indicating that these genes may play important roles in adaptation to aquatic environments. Notably, two of these genes were outstanding for their antioxidant role. One is the gene encoding NAT2 (Nucleobase-ascorbate transporter), which is involved in the ascorbate transport and the ascorbate play an important role in antioxidant [24] (Additional file 7). The other gene is *CAT2* encoding an enzyme Catalase-2, which is an anti-oxidant enzyme to protect cells from the toxic effects of hydrogen peroxide [25] (Additional file 7).

### Validation of the RNA-Seq data

To verify our RNA-Seq data, the expression levels of eight unigenes were examined by quantitative real-time PCR (qRT-PCR) experiment. Of the eight genes, two genes are known to encode Glutathione S-transferases (GST) and Glutathione peroxidases (GPX), which are antioxidant enzymes (Table 5); two genes encode Photosystem I subunit XI (PsaL) and Photosynthetic electron transport ferredoxin (PetF), which are associated with photosynthesis process (Table 5); four genes encode Alcohol dehydrogenase (ADH), Lactate dehydrogenase (LDH), Alanine aminotransferase (AlaAT) and Aspartate transaminase (AspAT), which are involved in carbohydrate metabolism, including fermentative pathway and a modified tricarboxylic acid cycle mode - alanine metabolism (Table 5). All eight genes exhibited higher expression level in response to flooding (Table 5), indicating that these genes are indeed stimulated by flooding stress. In addition, their expression changes measured from qRT-PCR were consistent with those estimated from the RNA-Seq data (Table 5).

**Table 5:** Validation of the DEGs with qRT-PCR experiment

Unigene ID	Gene name	Fold change by RNA-Seq	Fold change by qRT-PCR
d2.comp51231_c0_seq1	*GST*	8.79	10.85 ± 0.941
d2.comp347335_c0_seq1	*GPX*	6.23	5.23 ± 0.115
CL3838contig1	*ADH1*	2.29	1.74 ± 0.086
d2.comp60738_c0_seq1	*LDH*	6.07	3.47 ± 0.343
d2.comp51785_c0_seq1	*AlaAT*	7.91	9.82 ± 0.124
d2.comp63174_c0_seq1	*AspAT*	2.10	1.46 ± 0.047
d2.comp58507_c0_seq3	*PsaL*	7.94	11.60 ± 0.029
d2.comp47781_c0_seq1	*PetF*	6.23	3.92 ± 0.279

Note: The confirmation of expression level of candidate genes examined by real-time PCRs with three technical replications. *DEG* differentially expressed genes

## Discussion

In this study, using the RNA-Seq technology, transcriptome changes of *N. peltata* were obtained under both normal and flooding conditions. In total, 78,037,588 and 103,266,542 high-quality reads were acquired for US and TS sample, respectively. Gene annotation towards various databases (NR, COG, Swissprot, GO, and KEGG) was conducted after reads assembly. After calculation of gene expression for each unigene and a strict criterion, a total of 8883 unigenes were defined as DEGs, which were either induced or depressed by flooding stress. Besides, the top 20 ranked differentially expressed unigenes were mainly involved in antioxidant, photosynthesis and protein-related activity. In addition, GO enrichment and KEGG enrichment analysis of DEGs also presented processes involved in carbohydrate source (photosynthesis), self-protection area (antioxidants) and energy supplier (glycolysis). Moreover, the molecular adaptation of the aquatic plants compared with non-aquatic plants suggested that genes involved in anti-oxidant processes having significantly higher evolutionary rates in aquatic plants. These findings indicate that processes mentioned above play important roles in response of *N. peltata* to flooding.

When plants under flooding pressure, many physiological responses will be induced. Ethylene release was one of these responses. Previous studies showed that the shoot elongation strategy was mainly driven by ethylene, such as rice and *Rumex* [10, 22]. In *N. peltata*, the shoot elongation was also driven by ethylene and has been physiologically validated [13]. In addition, the cell number was increased to a large number, which contributed to the rapid elongation. Our transcriptome results identified some ethylene response factors and some cell cycle related genes such as *CDC7* and *CAFP*, which proved the fact that ethylene release was induced by flooding as a physiological response. Moreover, flooding often leads plants to oxygen shortage situation, which can induce ROS generation, and anti-oxidant enzymes were activated to protect the cell membrane [26, 27]. In *N. peltata*, though we were not able to detect enzyme activity, we can infer that the activity of antioxidant enzymes was induced under flooding stress. Several antioxidant genes such as *GST* and *GPX* and two fast evolving genes *NAT2* and *CAT2* involved in anti-oxidant were identified, indicating an activated ROS elimination process. Taken together, these results validated the physiological response of *N. peltata* under flooding stress.

Plants are challenged by various abiotic stresses. The increased accumulation of reactive oxygen species (ROS), including singlet oxygen, superoxide radicals, and hydrogen peroxide, is a key signature of abiotic stress at the molecular level [28]. To resist this harmful effect, plants employ a system that catalyzes the elimination of ROS and fights with oxidative damage via the formation of antioxidant enzymes, such as GST and GPX [29–31]. In the present study, the expression of DEGs encoding antioxidant enzymes GST and GPX was greatly larger than that under normal conditions, which has been validated by the qRT-PCR experiment (Table 5). Consistently, many antioxidant enzymes have been shown to be essential for plant survival during adaptive responses to waterlogging or flooding stress [22, 32–34]. Therefore, these antioxidant enzymes may be induced by flooding stress and critical for the survival of *N. peltata* in a submerged environment.

When plants live in a submerged environment, both light and carbon supplies are limited due to the slower diffusion rates in water [35, 36], which decreases plant photosynthesis performance. However, photosynthesis is important for plant survival, which makes the continuation of aerobic respiration through the elevated oxygen concentrations possible. The aerobic respiration is more efficient compared with anaerobic metabolism [37]. For *N. peltata*, transcriptome analysis revealed that many photosynthesis processes were predominantly enriched in GO terms (Fig. 2) and KEGG pathways (Fig. 3), suggesting the continuation of photosynthesis under flooding stress. Previous studies showed that submerged plants have physiological adaptation mechanisms termed carbon-concentrating mechanisms (CCMs) to produce more CO_2_ [38], including use of HCO_3_^−^ and crassulacean acid metabolism (CAM) [39, 40]. Moreover, the use of HCO_3_^−^ as a CO_2_ substrate is a common way for most true aquatic plants [41, 42]. Therefore, the continuation of photosynthesis of *N. peltata* under submergence may be attributed to the mechanism of using HCO3^−^. In addition, species that have leaf gas films or that can produce new leaves under waterlogging often have higher CO_2_ affinity and higher CO_2_ concentrations [43, 44]. Therefore, the similar ability of *N. peltata* to produce new acclimated leaves during submergence might also contribute to the continuation of photosynthesis underwater. Although the activation of photosynthesis was indicated by our petiole transcriptional data, further studies should be added to draw a reliable conclusion on photosynthesis of *N. peltata* under flooding stress.

Large amounts of energy and carbohydrate are required for the rapid submergence-induced petiole elongation of *N. peltata*. The initial response to a submergence environment is the induction of anaerobic metabolism [3]. For *N. peltata*, many DEGs involved in the fermentative pathway were identified and also the glycolysis process and pyruvate metabolism (ko00710) was predominantly in the GO and KEGG enrichment analysis (Figs. 2 and 3). The similar performance of these two enzymes stimulated by waterlogging has also been reported in other plants [23, 45], indicating that the fermentative pathway was likely activated to provide essential energy. However, this universal anoxia metabolism has a side effect: low efficiency [46]. Considering the active and rapid response of *N. peltata* to flooding stress, there should be alternative metabolic forms with high efficiency to supply the energy required. Moreover, in order to keep the efficiency in glycolysis under oxygen deficiency, it is important to remove accumulated pyruvate. The enzyme AlaAT (Alanine aminotransferase) can transform pyruvate into alanine, and increased alanine accumulation, which is correlated with enhanced activity of AlaAT, under anoxic conditions has been reported in other plants [47, 48]. This process is also likely present in *N. peltata* because DEGs encoding AlaAT were identified and their up-regulation expression level was validated by the qRT-PCR experiment (Table 5). In addition, a modified tricarboxylic acid (TCA) cycle mode-alanine metabolism was found to be induced by waterlogging in *Lotus japonicus* [49]. The yield produced from the metabolic reprogramming associated with alanine metabolism (4 ATP) doubled energy produced from glycolysis (2 ATP) [49]. Another DEGs encoding critical enzyme in alanine metabolism, AspAT (Aspartate transaminase), whose expression level was validated by the qRT-PCR, indicating that alanine metabolism was likely induced as energy source by flooding in *N. peltata*.

It is well known that ethylene is one of the main drivers for depth adaptation in flooding-tolerant plants [13]. Furthermore, group VII ethylene response factors (GVIIERFs) were identified to activate the expression of hypoxia-related genes by an N-end rule pathway under low oxygen conditions [50]. GVIIERF proteins were found in many plants such as *Arabidopsis thaliana*, *Oryza sativa* and *Rumex palustris* (Table 4). In *N. peltata*, we identified 3 GVIIERFs with the characteristic MCGGAIL amino-terminus in our transcriptome assembly. Indeed, GVIIERFs were found in other aquatic plants as *Ranunculus bungei* and *Utricularia gibba*, even can be found in the marine angiosperm *Zostera marina* by searching its genome sequence. These findings indicate that the oxygen sensing mechanism via GVIIERFs may be conserved in *N. peltata* and in other higher plants. Notably, we didn’t find GVIIERFs in the differential gene sets of *N. peltata*, possibly because of our long-duration sampling. Specifically, in this study we were mainly interested in the expression changes of unigenes at the time point of the seventh day, which may be too late to detect the expression changes of GVIIERFs. Indeed, the release of ethylene is an earlier signal in response to flooding [22].

## Conclusions

In the present study, comprehensive and valuable genomic resources were built by comparative transcriptome of petioles under normal and flooding conditions of *Nymphoides peltata*. Our data suggests that two processes rarely occurred in other flooding-tolerant plants, active photosynthesis and alanine metabolism, are likely contributed to the active response of *N. peltata* to flooding stress. These results deepen our understanding of the genetic basis underlying the response to flooding stress in aquatic plants. The response of plants to abiotic stresses is a complex network functioning with the regulation of stress-related genes [51], therefore, further investigations are still essential to detail the active responses of aquatic plants to flooding stress.

## Methods

### Plant growth and flooding treatment

Young, healthy *N. peltata* plants were identified by Professor Dan Yu and collected from Liangzi Lake (30°15′29″N, 114°33′30″E) and cultured in glass tanks in a greenhouse at Wuhan University, China. After a week of culturation, the experiment was conducted in May 2014. We chose 20 plants with a height of about 15 cm and transplanted them into two glass tanks, 10 in each tank. We added water in the two tanks to reach water depth of 15 cm and acclimatized plants two days. One tank was used as the untreated group keeping the water depth of 15 cm, the other served as the treated group increasing the water depth to 100 cm. The submerged leaves in the treated tank reached the water surface in the seventh day by elongation of their petioles. Then plant petioles were collected. Petioles from the control tank were used as untreated sample (US) and those from the treated tank were considered as treated sample (TS). Each sample was the mixture of petioles from 5 plants. Samples were frozen in liquid nitrogen and stored at −80 °C prior to RNA extraction. Total RNA of each sample was extracted using HiPure Plant RNA Kits (Magen, China) following the manufacturer’s instruction.

### Library construction and transcriptome sequencing

Sequencing was conducted commercially following the manufacturer’s instructions after checking the quality and concentration of RNAs. The procedure was as follows: fragmenting the mRNA after purification, synthesis of the first and second strand cDNA, and adding specific sequence adaptors. After that, cDNA fragments of ~200 bp were chosen to conduct with PCR amplification. The original image data were transferred into raw reads and saved as “fastq” files. The raw reads generated in this study have been deposited in NCBI database under accession number SRA259910.

The adapter sequences and low quality base calls were removed. Firstly, the pooled strategy was employed, the left files from both samples were mixed into left.fq file, and right files from both samples were also mixed into the right.fq file. Transcriptome assembly was accomplished based on the left.fq and right.fq using Trinity with default parameters with the pooled strategy [52]. Then separate assembly of sample US and sample TS were made using Trinity in order to prove our assembly quality. For both strategies, redundancy and over-representation were reduced after Trinity assemble by finding similar sequences using TIGR Gene Indices clustering program (TGICL) and Cluster database at high identity with tolerance (CD-HIT) with minimum 90 and 95% similarity cut off respectively [53, 54]. The TransDecoder was then used to identify the possible coding sequence (CDS) from the assembled sequences.

### Functional annotation of pooled unigenes

To gain a better comprehension of the transcriptom information, the pooled assembly unigene sequences was used since some unigenes with low expression levels generated with the pooled strategy cannot be found when using separate sample strategy due to their less reads in sample TS and sample US, which is an advantage of pooled strategy over separate assemble. These unigene sequences were used towards the Phytozome, NCBI NR, COG and Swissprot database, respectively, with a cutoff E-value of 1.0E-5. Only the best match gene ID was assigned to each unigene. Functional annotation by gene ontology analysis was analyzed by Blast2GO software with an E-value ≤1.0E-5. In addition, KEGG pathway analysis was conducted using the KEGG Automatic Annotation Server (KAAS).

### Identification of differentially expressed genes

To examine the expression level of each unigene in both samples, the expression of each unigene generated with the pooled strategy was calculated using the Cufflinks program [55]. Moreover, the unigene expression was normalized using the fragments per kilo bases per million reads (FPKM) method described by Mortazavi [56]. Subsequently, the differential gene expression between US and TS were analyzed using the edgeR software [57], with an FDR of 0.05 and |logFC| ≥ 1 as the threshold. DEGs were conducted GO enrichment analysis and KEGG enrichment analysis using R based on hypergeometric distribution. Significantly enriched GO terms and KEGG pathways were identified based on the corrected *P*-value (*P* < 0.01 and *P* < 0.05, respectively).

### Transcriptome changes of *Nymphoides peltata* and five other plants in response to flooding

To have a better understanding of the plants responses under flooding stress and to find the similarities/differences of waterlogging responses in *Nymphoides peltata* with other land plants, transcriptome comparisons among *Nymphoides peltata* and five other plants (*Arabidopsis thaliana*, *Oryza sativa*, *Rumex palustris*, *Lotus japonicas*, *Taxodium mucronatum* × *T. distichum*) were conducted. The transcriptome changes of other five plants were from previously published paper. In this method, candidate genes and biological processes involved in flooding response were compared across *Nymphoides peltata* and five other plants.

### Molecular adaptation of aquatic plants compared with non-aquatic plants

A comparative analysis between aquatic plants and non-aquatic plants was carried out to identify commonalities in aquatic plants. The genome of aquatic species *Utricularia gibba* was downloaded from https://genomevolution.org/CoGe (v4.1, ID 19475) and transcriptome reads of aquatic *Ranunculus bungei* were from SRR1822529 under the NCBI. Genomes of five non-aquatic plants including *Solanum lycopersicum* (assembly SL2.50), *Daucus carota* (assembly ASM162521v1), *Cucumis sativus* (assembly ASM407v2), *Oryza sativa* (assembly Build 4.0) were downloaded from NCBI and the genome sequences of *Arabidopsis thaliana* were from The *Arabidopsis* Information Resource (TAIR10). To identify one-to-one orthologous genes, the reciprocal BLAST approach was performed with each species’ CDS sequences and *Arabidopsis* proteins as query. E-value of 1e-5 was applied and the best hit was retained. The protein IDs of *Arabidopsis* were used as reference, a total of 5319 one-to-one orthologous in all species were identified and CDS sequences were extracted using a perl script. Each orthologous gene set was aligned used the PRANK program [58]. All of the genes were aligned at the codon level with the following settings: -shortnames +F -termgap -codon -f = fasta. Following alignment, Gblocks program was employed to identify the conserved regions at the codon level [59]. To detect fast evolving genes in aquatic plant group, we estimated a two-ratio branch model allowing different ω values (the ratios of nonsynonymous to synonymous substitution rates) in aquatic and non-aquatic plants and one-ratio model assuming a uniform ω value in all plants, using codeml in the PAML4.8 package [60]. In our analysis, we set the aquatic plants as the foreground, the other branches were set as the background. If a given gene was estimated to have a significantly higher in the foreground branches (ω_1_) than in the background branches (ω_0_) (corrected *p* < 0.05, FDR method), the gene would be considered as a candidate undergoing molecular adaptation.

### Validation of quantitative real-time PCR (qRT-PCR)

To validate the Illumina sequencing results, eight unigenes involved in those processes that may be responsible for the adaptation were selected for analysis using qRT-PCR. Gene-specific primers were designed with the Primer Premier 5.0 software, and these primer sequences can be found in Additional file 8. RT-PCR was performed as follows: 95 °C for 3 min, 35 cycles at 95 °C for 30 s, 54–64 °C for 30 s and 72 °C for 20 s; and final extension at 72 °C for 3 min. qRT-PCR was conducted using the SuperReal PreMix Plus (SYBR Green) (Tiangen Biotech, Beijing, China) and a CFX Real-Time PCR System (Bio-Rad, CA, USA). The actin like gene was used as internal reference controls to standardize the results. Statistical analysis was performed using the 2^- ΔΔCT^ method. The final values were presented as means of three independent biological trials.

## Additional files

Additional file 1: The distribution of sequence lengths for the unigenes predicted from the pooling transcriptome assembly of *Nymphoides peltata*. (PDF 122 kb)
Additional file 2: GO (Gene ontology) and COG (Cluster of orthologous groups) annotation for the pooling transcriptome assembly of *Nymphoides peltata*. (a) Functional annotation of the assembled unigenes based on GO category, the functions of the unigenes were divided into three categories. (b) Information of clusters of orthologous groups (COG) classification, the unigenes were mainly clustered into 25 components. (PDF 295 kb)
Additional file 3: Swissprot annotation for the total unigenes generated from the pooling transcriptome assembly. (XLS 4955 kb)
Additional file 4: The deduced protein sequences of all predicted genes in the pooling transcriptome assembly of *Nymphoides peltata*. (TXT 21193 kb)
Additional file 5: Unigene predictions from the *Nymphoides peltata* transcriptome assemblies based on three databases: Phytozome, NR (NCBI non-redundant proteins), and Non-coding RNA. (PDF 251 kb)
Additional file 6: This table provides all differentially expressed genes meeting the threshold (FDR ≤ 0.01) and the GO terms that the differentially expressed genes were enriched. (XLS 207 kb)
Additional file 7: Evolutionary analysis of molecular adaptation of aquatic plants compared with non-aquatic plants. (PDF 158 kb)
Additional file 8: Primer sequences used in the qRT-PCR experiment. (PDF 90 kb)

## Abbreviations

AlaAT: Alanine aminotransferase
AspAT: Aspartate transaminase
CAFP: Cell division cycle protein 48 homolog
CAT: Catalase-2
CCMs: Carbon-concentrating mechanisms
CDC7: Cell division cycle 7
COG: Cluster of orthologous groups
DEGs: Differentially expressed genes
FDR: False discovery rate
FPKM: Fragments per kilo bases per million reads
GO: Gene ontology
GPX: Peroxidase
GSH: Glutathione
GVIIERFs: Group VII ethylene response factors
KEGG: Kyoto encyclopedia of genes and genomes
NAT2: Nucleobase-ascorbate transporter 2
NR: NCBI non-redundant proteins
PCR: Polymerase chain reaction
PetF: Photosynthetic electron transport ferredoxin
PsaL: Photosystem I subunit XI
qRT-PCR: Quantitative real-time PCR
RNA-Seq: RNA sequencing
ROS: Reactive oxygen species
SRA: Sequence read archive
Swissprot: Swiss-prot protein sequence database
TS: Treated sample
US: Untreated sample
