# Supplementary material for: Transcriptome profiling of the floating-leaved aquatic plant Nymphoides peltata in response to flooding stress
Source: BMC Genomics. 2017 Jan 31;18:119. doi: 10.1186/s12864-017-3515-y (PMC5282827; doi:10.1186/s12864-017-3515-y)

Additional file 1: The distribution of sequence lengths for the unigenes predicted from the pooling transcriptome assembly of *Nymphoides peltata*.

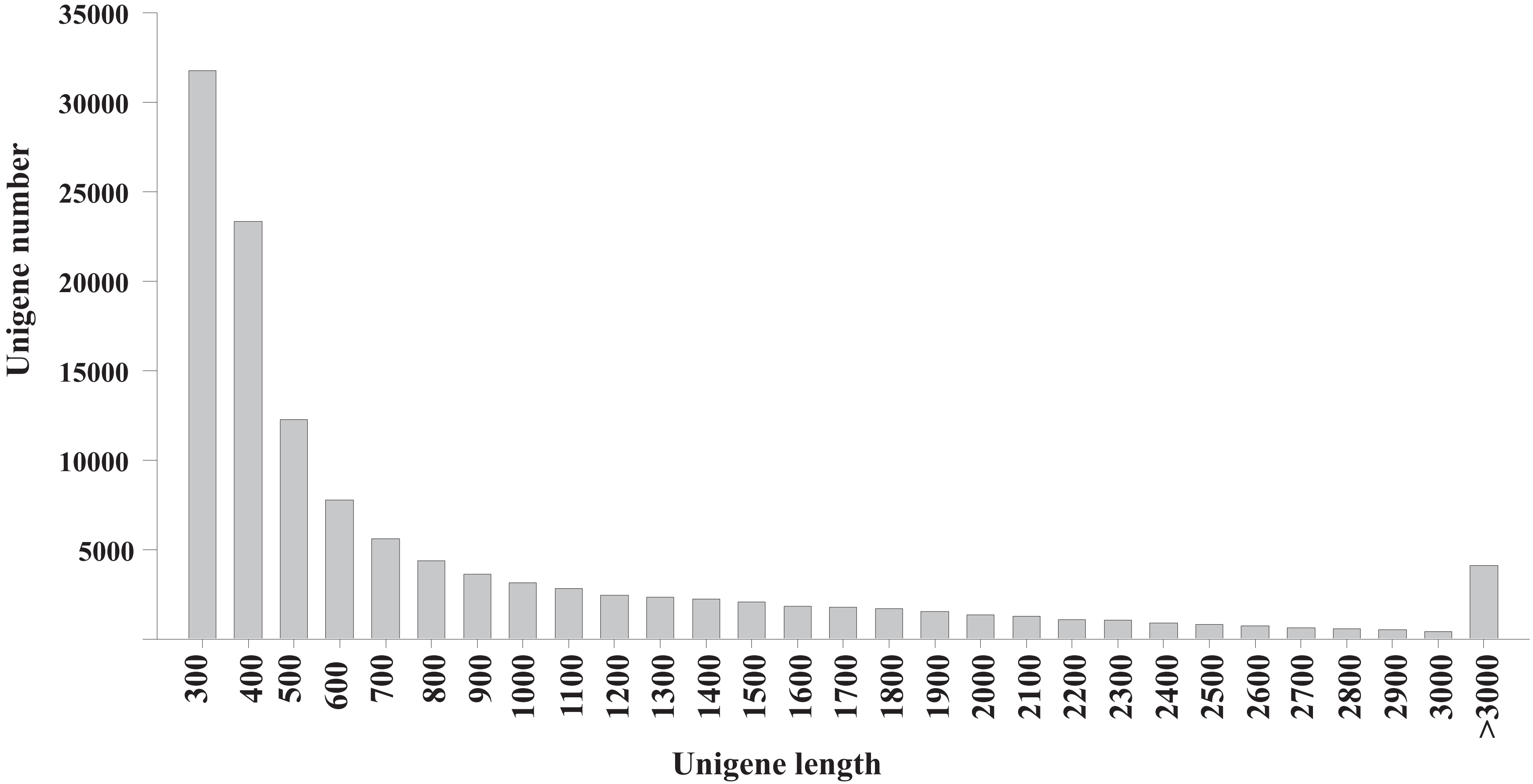

Supplement: Additional file 1: — The distribution of sequence lengths for the unigenes predicted from the pooling transcriptome assembly of Nymphoides peltata. (PDF 122 kb) [file 12864_2017_3515_MOESM1_ESM.pdf]
