# Supplementary material for: Transcriptome profiling of the floating-leaved aquatic plant Nymphoides peltata in response to flooding stress
Source: BMC Genomics. 2017 Jan 31;18:119. doi: 10.1186/s12864-017-3515-y (PMC5282827; doi:10.1186/s12864-017-3515-y)

Additional file 2 (a) Functional annotation of the assembled unigenes based on GO category, the functions of the unigenes were divided into three categories. (b) Information of clusters of orthologous groups (COG) classification, the unigenes were mainly clustered into 25 components.

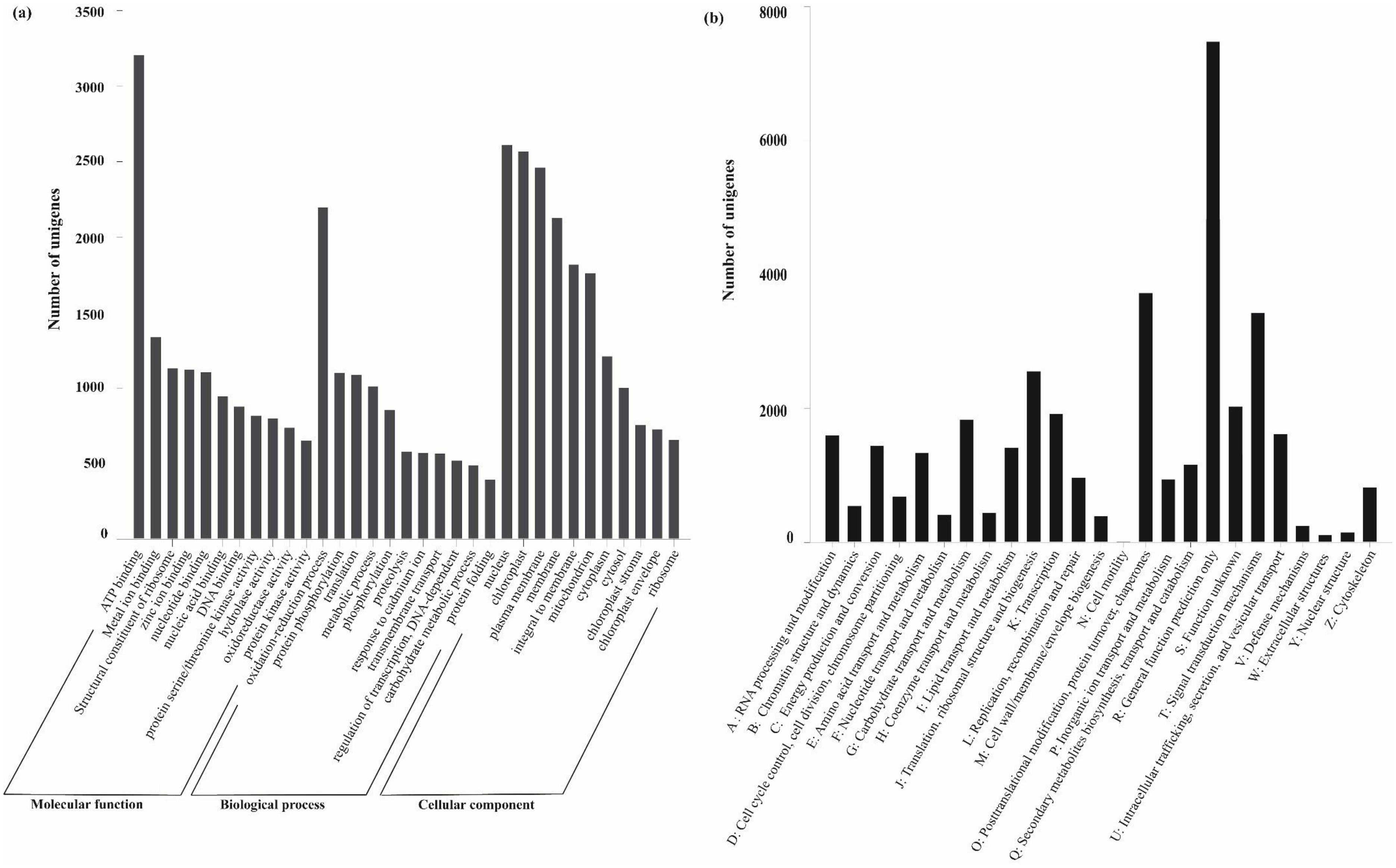

Supplement: Additional file 2: — GO (Gene ontology) and COG (Cluster of orthologous groups) annotation for the pooling transcriptome assembly of Nymphoides peltata. (a) Functional annotation of the assembled unigenes based on GO category, the functions of the unigenes were divided into three categories. (b) Information of clusters of orthologous groups (COG) classification, the unigenes were mainly clustered into 25 components. (PDF 295 kb) [file 12864_2017_3515_MOESM2_ESM.pdf]
