# Supplementary material for: Transcriptome profiling of the floating-leaved aquatic plant Nymphoides peltata in response to flooding stress
Source: BMC Genomics. 2017 Jan 31;18:119. doi: 10.1186/s12864-017-3515-y (PMC5282827; doi:10.1186/s12864-017-3515-y)
Supplement: Additional file 5: — Unigene predictions from the Nymphoides peltata transcriptome assemblies based on three databases: Phytozome, NR (NCBI non-redundant proteins), and Non-coding RNA. (PDF 251 kb) [file 12864_2017_3515_MOESM5_ESM.pdf]

Additional file 5. Unigene predictions from the *N. peltata* transcriptome assemblies based on three databases: Phytozome, NR (NCBI non-redundant proteins), and Non-coding RNA.

Category	Phytozome	NR	Unannotated	Predicted CDs	Non-coding RNA
US	37942	37697	49377	45973	58
TS	43183	42654	51745	48342	59
US+TS pool	52820	53744	69692	64753	43

Note: US means untreated sample, TS means treated sample. US+TS pool means the pooled sample using clean reads from treated and untreated sample. Predicted CDs mean the predicted coding gene number of the unannotated unigenes. Non-coding RNA indicates the number of unannotated unigenes in Phytozome and NR databases but annotated to the Non-coding RNA database.
