# Supplementary material for: Transcriptome profiling of the floating-leaved aquatic plant Nymphoides peltata in response to flooding stress
Source: BMC Genomics. 2017 Jan 31;18:119. doi: 10.1186/s12864-017-3515-y (PMC5282827; doi:10.1186/s12864-017-3515-y)
Supplement: Additional file 7: — Evolutionary analysis of molecular adaptation of aquatic plants compared with non-aquatic plants. (PDF 158 kb) [file 12864_2017_3515_MOESM7_ESM.pdf]

Additional file 7. Evolutionary analysis of molecular adaptation of aquatic plants compared with non-aquatic plants.

Unigene ID	Gene name	ω of aquatic plants	ω of non-aquatic plants	*P* value
TR6629_c1_g1_i1	*ABCB19*	0.036874	0.023494	0.028
TR10093_c0_g1_i2	*HEMB1*	0.059597	0.027808	0.039
TR60631_c0_g1_i1	*ABCB1*	0.076195	0.036114	5.24E-06
TR44321_c0_g1_i1	*RAD50*	0.132137	0.079645	0.028
CL3482Contig1	*CCR3*	0.06995	0.024871	0.027
TR21240_c1_g1_i2	*ATJ13*	0.157833	0.098019	0.033
CL680Contig1	*SPE2*	0.056426	0.023564	0.033
TR977_c2_g1_i1	*At4g23740*	0.121856	0.048377	0.026
TR15463_c0_g1_i1	*NAT2*	0.110638	0.044651	0.007
TR47905_c0_g1_i5	*STN8*	0.092683	0.038917	0.016
CL4684Contig1	*DXS*	0.05216	0.025734	0.007
TR25987_c0_g1_i2	*LNG2*	0.221001	0.13413	0.026
TR29764_c0_g1_i3	*At2g20050*	0.122199	0.053947	2.62E-05
TR52493_c0_g1_i2	*CAT2*	0.05629	0.028676	0.018
TR31717_c0_g1_i3	unknown	0.352998	0.072004	0.008
TR61766_c0_g1_i12	unknown	0.170641	0.080018	0.015
CL6880Contig1	unknown	0.161334	0.020046	0.015
TR49871_c1_g2_i2	unknown	0.082749	0.021676	0.035

Note: *P* value indicated the *P* value corrected by false discovery rate at 5%. ω means nonsynonymous to synonymous substitution rates
