# Supplementary material for: Transcriptome profiling of the floating-leaved aquatic plant Nymphoides peltata in response to flooding stress
Source: BMC Genomics. 2017 Jan 31;18:119. doi: 10.1186/s12864-017-3515-y (PMC5282827; doi:10.1186/s12864-017-3515-y)
Supplement: Additional file 8: — Primer sequences used in the qRT-PCR experiment. (PDF 90 kb) [file 12864_2017_3515_MOESM8_ESM.pdf]

Additional file 8. Primer sequences used in the qRT-PCR experiment.

Unigene ID	Gene name	Abbr.	Sequences (5' -3')
d2.comp51231_c0_seq1	glutathione S-transferase	*GST*	GGTCGAACCTGACTGGGAAGA
			GGGCGTTGGGAAAGAATGG
d2.comp347335_c0_seq1	glutathione peroxidase	*GPX*	GGTTGGACGCTTGTTCATCAC
			CGTTTGAGCCCTGTATCTTTG
CL3838contig1	alcohol dehydrogenase	*ADH*	CAACCCACTTGCTCCTCT
			CCCTGAAACTCTAGCTCCTT
d2.comp60738_c0_seq1	lactate dehydrogenase	*LDH*	GAACTTCTTAGATAGGGAGCAA
			AGAACACTTCGCCATCGT
d2.comp51785_c0_seq1	alanine aminotransferase	*AlaAT*	GGAACCTTGGTGCCTTATT
			TTCTTGGTACACCTCGTCG
d2.comp63174_c0_seq1	aspartate transaminase	*AspAT*	AAGCTGAGCGGAGGATTG
			AACTGGGATGTAGATTTGTGA
d2.comp58507_c0_seq3	photosystemI subunit XI	*PsaL*	GCTGCCGAGAAGTATCAAGTCA
			GTATCCGTGAGCCAGACCAAC
d2.comp47781_c0_seq1	photosynthethic electron transport ferredoxin	*PetF*	ACGACACGTACATCCTCGACG
			GGAGATGGTGCAGTCGGAGGTG
con2.comp52638_c0_seq3	actin-like protein	*Actin*	CTGTCGAAATGAGGCTGAA
			GCAACTCTTCGCAATGGTA

Note: Sense means sense primer, Anti-sense means Anti-sense primer. Unigene con2.comp52638_c0_seq3 (i.e. actin-like gene) was used as an internal control to standardize the results. Abbr. represents abbreviation.
